# Self-Efficacy and Professional Identity Among Freshmen Nursing Students: A Latent Profile and Moderated Mediation Analysis

**DOI:** 10.3389/fpsyg.2022.779986

**Published:** 2022-03-03

**Authors:** Xiao Xiao Mei, Hui Yuan Wang, Xiao Na Wu, Jie Yi Wu, Ying Zi Lu, Zeng Jie Ye

**Affiliations:** School of Nursing, Guangzhou University of Chinese Medicine, Guangzhou, China

**Keywords:** self-efficacy, resilience, professional identity, moderated mediation analysis, latent profile analysis, freshmen nursing students

## Abstract

This study was designed to estimate the associations between self-efficacy and professional identity. A total of 1,051 freshmen nursing students (FNSs) from the Be Resilient to Nursing Career (BRNC) program were recruited from four universities between September and November 2020. A latent profile and moderated meditation analysis were performed. Four profiles of self-efficacy were identified and named as Lowest (15.6%), Med-low (45.0%), Med-high (32.7%), and Highest (6.7%). The mediating role of resilience and the moderating effect of role models were also identified. Therefore, self-efficacy, resilience, and role models may be three important factors to professional identity in FNSs and these relationships should be further validated in longitudinal or interventional studies.

## Introduction

The shortage of nurses has become a global issue and nursing students’ retention deserves additional attention from nursing scientists (Bakker et al., 2018; World Health Organization, 2020). Professional identity, defined as one’s view about professional attributes, beliefs, values, motive, and experience (Slay and Smith, 2010), is developed to increase the retention rate which has been confirmed as a key predictor for nurses’ retention (Guo et al., 2018; Wu et al., 2019). Ohlen and Segesten (1998) divide nurses’ professional identity into three dimensions: personal (personal confidence), interpersonal (interaction between nurses and other nurses, forming professional identity from nursing skills and knowledge), and social history (understanding nurses’ professional identity with social history). Professional identity develops through their understanding of the intended professional skills, qualities, conduct, culture, and ideology about nursing (Jackson, 2016; Browne et al., 2018). In addition, professional identity is positively associated with students’ mental health, clinical performance, and care quality (Hensel and Laux, 2014; Sun et al., 2016; Nyirenda and Mukwato, 2017; Wang et al., 2019). Thus, the methods of improving professional identity resulting in a high retention rate should be further explained, and intervention programs designed to improve freshman nursing students’ (FNS) professional identity may be cost-effective.

Self-efficacy, defined as people’s self-confidence in facing great challenges and breaking through the difficulties, may directly affect one’s professional identity (Bandura, 1997; Xiong et al., 2020). Low self-efficacy may cause burnout and low job satisfaction (Bulfone et al., 2016; Yao et al., 2018), resulting in a low professional identity level (Ren et al., 2021). Furthermore, self-efficacy was positively correlated with professional identity in the previous studies (Qiu et al., 2019; Shen et al., 2020).

In addition, self-efficacy plays a significant role in fostering resilience (Kumfer, 1999; Cody, 2013; Rees et al., 2015), which has been confirmed as the strongest predictor of professional identity (Bhattarai et al., 2020; Zhang et al., 2021). However, no empirical study has examined the mediating role of resilience in the relationship between self-efficacy and professional identity (Hayat et al., 2021; Ma et al., 2021). Lastly, role models play an important role in the development of professional identity in FNS (Paice, 2002; Sternszus et al., 2020). Role models show more possibilities and values of the nursing profession through their own career development, so as to enhance FNSs’ professional identity (Morgenroth et al., 2015; Wang, 2019). Thus, this variable should be further explained.

**FIGURE 1:**
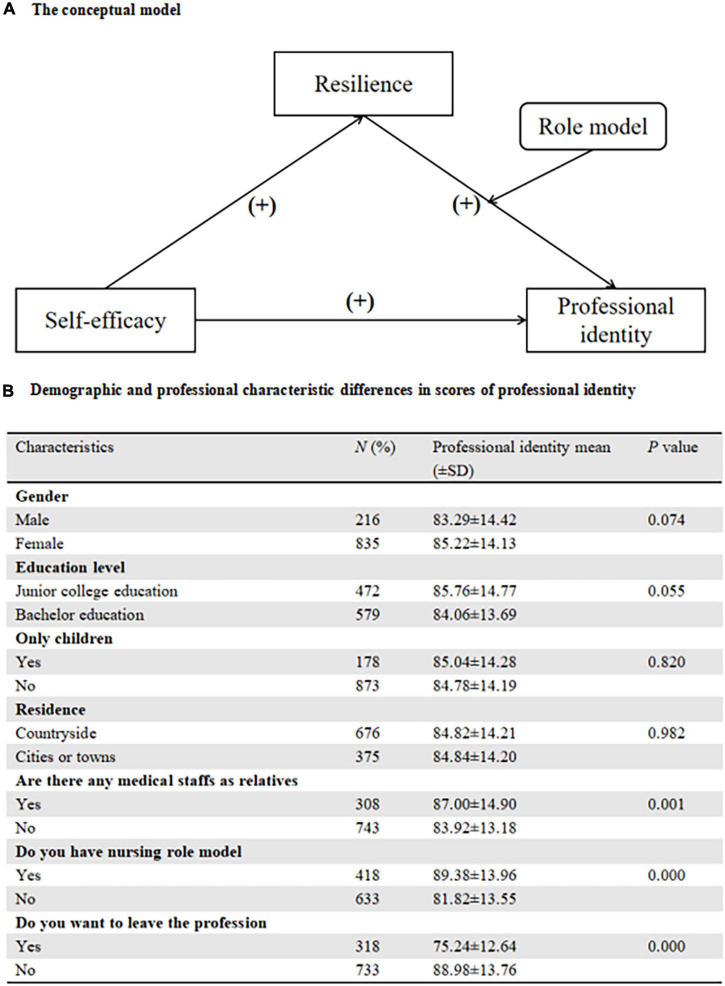
**(A)** The Conceptual model. **(B)** Demographic and professional characteristic differences in scores of professional identity.

To be brief, self-efficacy, resilience, and role models have been confirmed as potential factors to professional identity (Chen et al., 2020; Lyu et al., 2020; Sternszus et al., 2020) while the associations between self-efficacy, resilience, role models, and professional identity combined in one equation have not been fully explained. The current study was designed to fill the gap, including the mediation effects of resilience and the moderation effect of role models. Based on previous studies, we hypothesized that (Figure 1A):

H1: Self-efficacy would be a significant predictor of professional identity.
H2: Several distinct self-efficacy patterns would be identified by Latent Profile Analysis (LPA).
H3: Resilience may play a mediating role between self-efficacy and professional identity.
H4: Role models may play a moderate role in the resilience-professional identity linkage.

## Materials and Methods

### Participants

A total of 1,220 FNSs from four universities in Guangdong Province and Shandong Province were enrolled between September and November 2020. The inclusion criteria were as follows: (1) newly enrolled nursing students in 2020; (2) could communicate fluently in Mandarin; (3) willing to participate in this study. The exclusion criteria were: (1) had a diagnosis of mental disorders. All participants were approached by a researcher who verbally explained the purpose and procedure, and informed consent was obtained. Then, the instruments were administered and collected. The required minimum sample size was calculated, using the Power Analysis and Sample Size Software (PASS, version 15.0) (NCSS, LLC. Kaysville, UT, United States), with the confidence level of 95%, the distance from mean to limit of 1.5, and an SD of 15 (Bujang and Adnan, 2016). The minimum sample size required was 482 with a 20% dropout rate.

### Instruments

#### Demographics

Based on previous literature (Mussi et al., 2019; Xie et al., 2020), we collected the FNSs’ demographic (age, gender, educational backgrounds, place of residence) and profession-related information (do you have a nursing role model, are there any medical staffs as relatives).

#### Self-Efficacy

The General Self-efficacy Scale (GSES) was developed by Zhang and Schwarzer (1995), and the Chinese version has been proved to be reliable (Wang et al., 2001; Zhang et al., 2018). It has 10 items and was a 4-point-Likert scale ranging from 10 to 40. The scale is divided into three levels: low (10–20 points), medium (21–30 points), and high (31–40 points). The Cronbach’s alpha for GSES was 0.900.

#### Resilience

The Connor-Davidson Resilience Scale (CD-RISC) was developed by Connor in 2003, which consisted of 25 items (Connor and Davidson, 2003). A 10-item unidimensional version was later developed by Campbell-Sills and Stein (Campbell-Sills and Stein, 2007). It was a 5-Likert scale (0–4) with high scores indicating high resilience levels. The Chinese version of CD-RISC-10 has been approved to be reliable (Ye et al., 2017). The Cronbach’s alpha coefficient for CD-RISC-10 in this study was 0.874.

#### Professional Identity

The Chinese Version of the Professional Identity Questionnaire for Undergraduate Students (PIQUS) was developed by Qin (Qin, 2009). PIQUS has 23 items and includes four dimensions: cognitive identity, emotional identity, behavioral identity, and fit identity. It is a 5-Likert scale (1–5) with high scores indicating a high level of professional identity. In this study, the Cronbach’s alpha coefficients are 0.854 for cognitive identity, 0.914 for emotional identity, 0.855 for behavioral identity, and 0.858 for fit identity.

#### Data Analysis

First, descriptive analysis was used to describe the demographics and profession-related characteristics. In addition, Pearson’s correlation analysis was employed to determine the relationships among self-efficacy, resilience, and professional identity.

Second, LPA was performed to recognize the latent subgroups of FNSs’ self-efficacy. It began with a one-class model, continuing until fit indices could not be improved. For determining the number of latent groups, Akaike Information Criterion (AIC) (Simpson et al., 2018), Bayesian Information Criterion (BIC) (Coulombe et al., 2016), and sample size-adjusted BIC (ssaBIC) (Sclove, 1987) were utilized as indicators. The Lo–Mendell–Rubin likelihood ratio test (LMRT) and the bootstrapped likelihood ratio test (BLRT) (Lo et al., 2001) were used to test whether the introduction of one more category (i.e., latent group) contributed to a significant change compared to the previously derived model. ANOVA was applied to compare the professional identity among the FNSs with different self-efficacy profiles.

Third, Harman’s one-factor model was performed to verify the potential existence of the common method variance (CMV). The mediator role of resilience was first estimated between LPA-based self-efficacy profiles (category variable) and professional identity (including four domains) through the PROCESS macro (Model 4) of SPSS (IBM Corp., Armonk, NY, United States). Subsequently, resilience was enrolled into the regressions between self-efficacy (continuous variable) and professional identity and the effect of the role model was fully explained by PROCESS macro (Model 58). To be brief, self-efficacy was the independent variable (X), four domains of professional identity were the dependent variables (Y), resilience was the mediator, and role model was the moderated variable. The total, direct and indirect effects were estimated and the mediating effect was considered statistically significant if the 95% bootstrap confidence interval did not contain zero (Hayes, 2017). SPSS (IBM Corp., Armonk, NY, United States) (version 26.0) and Mplus (Muthén and Muthén, 2000) (version 8.3) were used for all statistical analyses.

## Results

### Sample Characteristics

A total of 1,220 FNSs were invited to participate in the survey but 169 participants were excluded because they refused to complete the survey (*N* = 93) or had missing data (*N* = 76). No significant demographic difference was identified between the included and the excluded (*P* > 0.05). The sex ratio of males to females was 1:3.87. In total, 54.8% FNSs had a bachelor education level and 64.3% FNSs were from the countryside. In this study, the mean score of professional identity was 84.82 ± 14.20. Significant differences of professional identity were identified in variables, including: are there any medical staff as relatives (*P* = 0.001), do you have a nursing role model (*P* < 0.001), do you want to leave the profession (*P* < 0.001). Other details were summarized in Figure 1B.

### Latent Profile Analysis of Self-Efficacy

**FIGURE 2:**
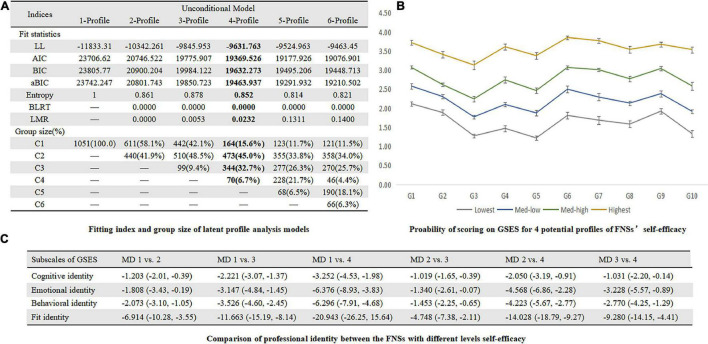
**(A)** Fitting index and group size of latent profile analysis models. **(B)** Probability of scoring on GSES for 4 profiles of FNS’s self-efficacy. **(C)** Comparison of professional identity between the FNSs with different levels self-efficacy.

In Figure 2A, LMRT indicated that a three-profile model was better than a two-profile one (*P* = 0.0053), and a four-profile model is better than a three-profile one (*P* = 0.0232) while the difference between a four-profile model and a five-profile one was not significant (*P* = 0.1311). Thus, according to the parsimonious guideline, we finally chose the four-profile solution. The subgroups were named as Lowest (15.6%), Med-low (45.0%), Med-high (32.7%), and Highest (6.7%) groups and other information was summarized in Figure 2B. The professional identity levels in different self-efficacy profiles were presented in Figure 2C.

### Meditation Analysis of Resilience Between Self-Efficacy Profiles (Category Variable) and Professional Identity

**FIGURE 3:**
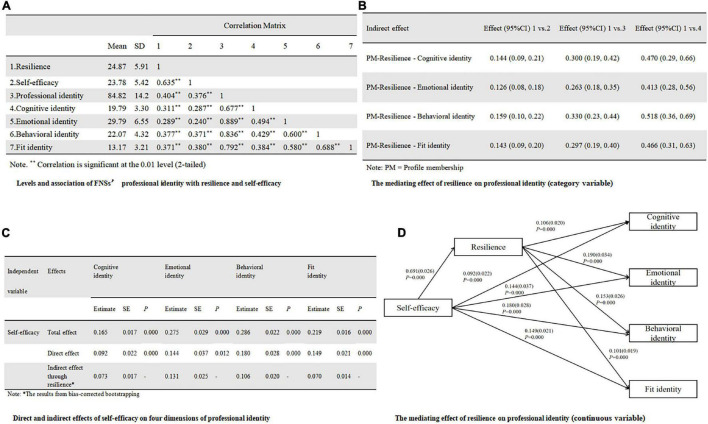
**(A)** Levels and association of FNS’s professional identity with resilience and self-efficacy. **(B)** The mediating effect of resilience on Professional identity (category variable). **(C)** Direct and indirect effects of self-efficacy on four dimensions of professional identity. **(D)** The mediating effect of resilience on professional identity (continuous variable).

The variance explained by the first factor was 30.3% and did not reach 50%, so the common method bias was controllable. The professional identity, GSES, and CD-RISC-10 were 84.82 (SD, 14.20), 23.78 (SD, 5.42), and 24.87 (SD, 5.91), respectively. Furthermore, professional identity was significantly correlated with self-efficacy (*r* = 0.376, *P* < 0.01) and resilience (*r* = 0.404, *P* < 0.01). Other information was summarized in Figure 3A. In Figure 3B, taking Profile 1 as the reference, the mediating effect of resilience between profile 2, profile 3, profile 4, and cognitive identity were 0.144, 0.300, and 0.470, respectively, with 95% Bootstrap confidence intervals of (0.09, 0.21), (0.19, 0.42), (0.29, 0.66), excluding “0,” indicating a significant mediating effect. Similar findings were identified in domains of emotional identity, behavioral identity, and fit identity in Figure 3B, indicating that resilience did play a mediating role between self-efficacy profiles and professional identity.

### Meditation Analysis of Resilience Between Self-Efficacy (Continuous Variable) and Professional Identity

Professional identity varied significantly on three demographic variables and these three variables were dummy coded and assigned as covariates in the regressions.

In Figure 3C, the significant total effect of self-efficacy were identified on all domains of professional identity, including: cognitive identity (*B* = 0.165, *SE* = 0.017, *P* < 0.001), emotional identity (*B* = 0.275, *SE* = 0.029, *P* < 0.001), behavioral identity (*B* = 0.286, *SE* = 0.022, *P* < 0.001), and fit identity (*B* = 0.219, *SE* = 0.016, *P* < 0.001). In Figure 3D, the significant mediation effect of resilience from self-efficacy to professional identity were also recognized, including: cognitive identity (*B* = 0.073, SEBoot = 0.017, 95%CI:0.04, 0.11), emotional identity (*B* = 0.131, SEBoot = 0.025, 95%CI:0.08, 0.18), behavioral identity (*B* = 0.106, SEBoot = 0.020, 95%CI:0.07, 0.15), and fit identity (*B* = 0.070, SEBoot = 0.014, 95% CI:0.04, 0.10).

### The Moderated Meditation Analysis Combing Resilience and Role Model

**FIGURE 4:**
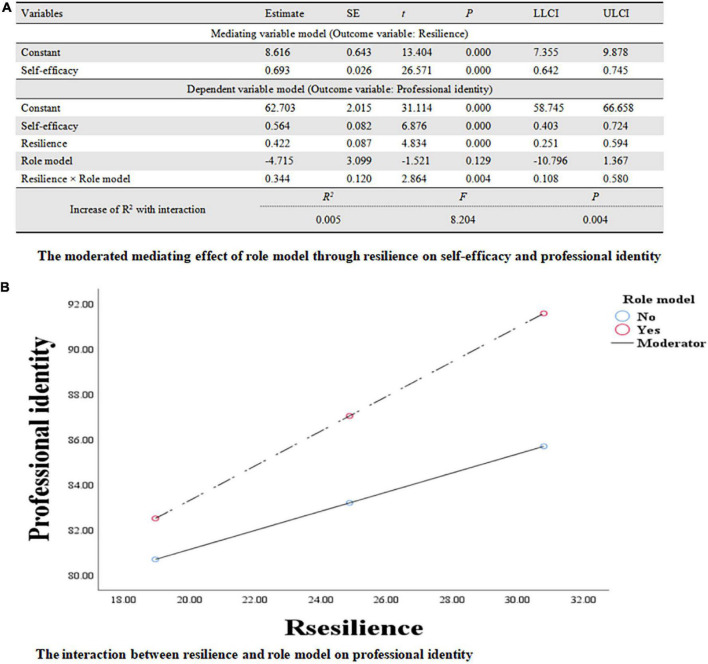
**(A)** The moderated mediating effect of role model through resilience on self-efficacy and professional identity. **(B)** The interaction between resilience and role model on professional identity.

In Figure 4A, the significant interaction between resilience and role model was recognized (*B* = 0.344, *SE* = 0.120, 95%CI:0.11, 0.58, *P* < 0.01), indicating that the relationship between resilience and professional identity was moderated by role models (*R*^2^ = 0.396, *F* = 113.821, *P* < 0.01). According to the simple slopes tests in Figure 4B, FNSs with role models (*B* = 0.766, *SE* = 0.106, 95%CI:0.56, 0.98, *t* = 7.201, *P* < 0.001) will have more professional identity increment compared to those without role models (*B* = 0.422, *SE* = 0.087, 95%CI:0.25, 0.60, *t* = 4.834, *P* < 0.001) when resilience was increased.

## Discussion

There existed heterogeneity in self-efficacy among FNSs and resilience could mediate the associations between self-efficacy and professional identity after controlling the effect of covariates. In other words, self-efficacy might promote individuals’ resilience resulting in improved professional identity. In addition, role models might moderate the associations between resilience and professional identity.

First, in this study, self-efficacy was positively associated with professional identity, which was consistent with previous research (Qiu et al., 2019; Eren and Turkmen, 2020). In addition, LPA was utilized to identify latent profiles of self-efficacy among FNSs and yielded a four-profile solution, which was consistent with Newnham et al. (2017) research. What’s more, both self-efficacy profiles (category variable) and self-efficacy (continuous variable) were significantly mediated by resilience in the current study, and how to enhance resilience resulting in higher levels of professional identity should be further explored. According to Stephens Model of Nursing Student Resilience (Stephens, 2013), as nursing students learn to identify, enhance, and/or develop their protective factors, they will be better equipped to effectively manage perceived adversity and stress. Cultivating resilience among nursing students may help with academic success, sustaining students’ demands for the nursing profession, and increasing professional identity (Smith and Yang, 2017; Keener et al., 2021; Zhang et al., 2021). These findings suggested that it was feasible to incorporate intervention techniques targeting resilience into professional identity enhancement programs for FNSs with low self-efficacy (Morrish et al., 2017; Lopez et al., 2018). For example, Chow et al. (2020) incorporated a resilience-building module into the undergraduate nursing curriculum for 195 nursing students and received a good response from the participants.

Second, role model was confirmed as a moderator between resilience and professional identity. In other words, the correlations between resilience and professional identity were stronger in FNSs with role models compared to those without role models. Role models were critically important in the professional, character, and career development of the medical students (Passi and Johnson, 2016; Mohamed Osama and Gallagher, 2018). For example, undergraduate nursing students valued role models as beneficial to their learning (Jack et al., 2017). In addition, role models could motivate nursing students to change their prejudices about the nursing profession through imitative learning, and allow FNSs to see more possibilities in the nursing profession, which in turn amplified the positive effect of resilience on professional identity. Thus, role models could be incorporated to enhance professional identity.

To conclude, this study contributed to better understanding the associations between self-efficacy and resilience and role model and professional identity among FNSs. Our results showed that (1) professional identity could be increased by self-efficacy and resilience; (2) role models could enhance the strength between resilience and professional identity.

## Limitations

Several limitations should be noted. First, the sample collected is based on Chinese FNSs, which may be different from western countries. Thus, the findings in the current study cannot be generalized to other FNSs with different backgrounds. Second, this study is cross-sectional and no causal relationships can be concluded. Longitudinal or interventional studies should be performed in the future to confirm these associations derived from the current study. Third, there still exists other factors affecting the professional identity, and more factors should be incorporated in future research with a more sophisticated method, for example, Structural Equation Model (SEM), which can take measurement bias into consideration and provide a more precise parameter estimation.

## Conclusion

Self-efficacy, resilience, and role models may be three important factors to professional identity in FNSs and these relationships should be further validated in longitudinal or interventional studies.

## Data Availability Statement

The raw data supporting the conclusions of this article will be made available from the corresponding author by request.

## Ethics Statement

The studies involving human participants were reviewed and approved by the Ethics Committee of The First Affiliated Hospital of Guangzhou University of Chinese Medicine [No: ZYYEC-ERK(2020)132]. The patients/participants provided their written informed consent to participate in this study.

## Author Contributions

XM: conceptualization, data curation, methodology, software, and writing—original draft. HW: investigation, resources, software, and validation. XW: investigation, software, and methodology. JW and YL: investigation and resources. ZY: supervision and writing—review and editing. All authors contributed to the article and approved the submitted version.

## Conflict of Interest

The authors declare that the research was conducted in the absence of any commercial or financial relationships that could be construed as a potential conflict of interest.

## Publisher’s Note

All claims expressed in this article are solely those of the authors and do not necessarily represent those of their affiliated organizations, or those of the publisher, the editors and the reviewers. Any product that may be evaluated in this article, or claim that may be made by its manufacturer, is not guaranteed or endorsed by the publisher.
